# Intestinal and Mucosal Microbiome Response to Oral Challenge of Enterotoxigenic *Escherichia coli* in Weaned Pigs

**DOI:** 10.3390/pathogens11020160

**Published:** 2022-01-26

**Authors:** Shan-Shan Peng, Yingjie Li, Qiuhong Chen, Qi Hu, Ying He, Lianqiang Che, Ping-Ping Jiang

**Affiliations:** 1 School of Public Health, Sun Yat-sen University, Guangzhou 510080, China; pengshsh6@mail2.sysu.edu.cn; 2 Key Laboratory for Animal Disease Resistance and Nutrition of the Ministry of Education, Institute of Animal Nutrition, Sichuan Agricultural University, Chengdu 611130, China; liyingjie@stu.sicau.edu.cn (Y.L.); 2020214006@stu.sicau.edu.cn (Q.C.); 2018214004@stu.sicau.edu.cn (Y.H.); 3 The Neomics Institute, Shenzhen 518122, China; huqi@neomics.org.cn; 4 Guangdong Provincial Key Laboratory of Food, Nutrition and Health, Sun Yat-sen University, Guangzhou 510080, China

**Keywords:** infant diarrhoea, enterotoxigenic *Escherichia coli*, proteomics, mucosal microbiome, regularised canonical correlation analysis, bile acid

## Abstract

Enterotoxigenic *Escherichia coli* (ETEC) is closely associated with diarrhoea in children in resource-limited countries. This study aims to investigate the change of the mucosal microbiome and protein expression in the ileum induced by *E. coli* K88 (ETEC) using pigs as a model. Seven weaned male pigs were orally given ETEC (1 × 10^9^ CFU, *n* = 7), and the other seven received saline (CON, *n* = 7). Ileal tissues were obtained 48 hours after the ETEC challenge for both proteomic and mucosal microbiome analyses. Nine proteins were found with altered abundance between the two groups, including a decrease in FABP1 and FABP6, involved in bile acid circulation. The TLR-9 mediated pathway was also affected showing increased transcription of genes SIGIRR and MyD88. Correlations between the ileal proteins and mucosal bacterial taxa found included a positive correlation between Lactobacilllus and PPP3CA (r = 0.9, *p* < 0.001) and a negative correlation between Prevotella with CTNND1 (r = −0.7, *p* < 0.01). In conclusion, ETEC infection caused inflammation and impaired the circulation of bile acids and the mucosal microbiome may affect the expression of intestinal proteins. Further studies are needed to explain the exact roles of these affected processes in the pathogenesis of ETEC-triggered diarrhoea.

## 1. Introduction

Diarrhoea is still one of the major causes of morbidity and mortality in children. Approximately 500,000 children died from diarrhoea in 2018, 20% of which happened in the second half-year of life [1,2,3]. Enterotoxigenic *Escherichia coli* (ETEC) is a major cause of diarrhoea in children in resource-limited countries [4]. ETECs adhere to the intestinal epithelial cells (IEC) in the jejunum and ileum through adhesins interacting with specific receptors and secret enterotoxins to perturb hydroelectrolytic secretions, resulting in rapid onset of secretory diarrhoea leading to dehydration [5].

Despite the high prevalence of ETEC-associated diarrhoea, the detailed response of the gut to ETEC infection is not yet fully understood. Multiple biological pathways and the gut microbiome were associated with ETEC-triggered diarrhoea by in vitro [6,7], ex vivo [8] and in vivo studies using pigs [9] and mice [10] as models. Affected pathways include an innate immune response involving nuclear factor-κB (NF-κB) and mitogen-activated protein kinase (MAPK), intestinal barrier function, intestinal ion transporters and the water channel [11]. Previous studies mainly focused on the intestinal response in the jejunal segment leaving that of the ileal segment less characterised. Intervention using probiotics, such as Lactobacilli [6,7] or *Saccharomyces cerevisiae* [12] highlights the involvement of the gut microbiome in ETEC-associated diarrhoea. Since an altered gut microbiome was also found in osmotic diarrhoea, the relationship between the changed gut microbiome and infectious diarrhoea remains unclear. Currently available studies on the gut microbiome are mainly on the cecum [13], jejunal digesta and faeces [14] of ETEC-treated animals. It is of interest to know how the mucosal microbiome changes post-ETEC infection. The mucosal microbiome refers to the bacteria that settle on the intestinal mucosa and is an important part of the gut microbiota. The mucosal microbiome is vital in blocking and delaying the translocation and infection of various pathogenic microorganisms. Compared with the luminal microbiome, the mucosal one may be relatively stable. Moreover, compared to the luminal microbiome, which assists digestion, the mucosal microbiome plays an important role in symbiosis with the hosting organism [15].

Herein this study, changes of ileal proteins and the mucosal microbiome in the ileum post-oral ETEC challenge in weaned pigs were profiled by untargeted proteomics and 16S amplicon sequencing, respectively. Correlation analysis was also conducted to explore the potential association between intestinal proteins and bacterial taxa.

## 2. Results

Weaned male pigs (aged 28 d) were randomly allocated into two groups, one received ETEC challenge (the ETEC group, *n* = 7) and the other group received sterile carrier as the CON group (*n* = 7). Each group was reared in one pen.

### 2.1. Body Weights, Diarrhoea Scores and Ileal Morphology

**Figure 1:**
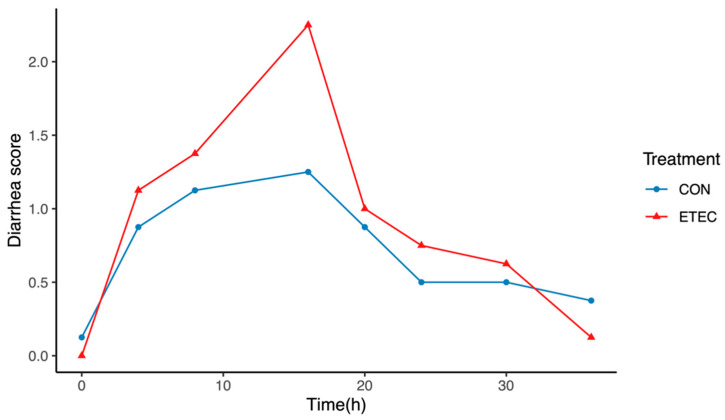
The diarrhoea score over time post-ETEC challenge.

The diarrhoea scores were recorded for each rearing pen where the pigs were reared over the time post-ETEC challenge, thus no SD can be calculated, and are shown in Figure 1. The difference in the diarrhoea scores peaked at 16 h between the two treatment groups. At 36 h post-challenge, the diarrhoea scores in the ETEC group were close to those in the CON group. No significant difference was observed in the body weight between the ETEC and CON pigs at euthanasia (*p* > 0.05). No significant difference was observed in intestinal morphological parameters in the ileum, such as the villous height, the crypt depth or the ratio of villous height over crypt depth between the two treatment groups (*p* > 0.05). The body weight at euthanasia and the intestinal morphological parameters are presented in Table 1.

### 2.2. Ileal Proteomics

In total, 5151 ileal proteins were annotated by LC-MS-based proteomics and 47 proteins were selected by the semi-multivariate analysis, Nearest Shrunken Centroid (NSC) analysis, as being able to differentiate the two treatment groups. Nine proteins had significantly different abundance between the ETEC and CON pigs (*p* < 0.05, |effect size| > 0.80) as further tested by Student’s *t*-test. Information on these proteins, including accession number, protein name, gene name, abundance in the two treatment groups, *p*-value and effect size and biological process are listed in Table 2. Four proteins showed lower abundance in the ETEC pigs relative to the CON ones, including fatty acid-binding proteins 6 (FABP6, the ileal form) and 1 (FABP1, the liver form), ADP ribosylation factors such as GTPase 8B (ARL8B) and Cytochrome C oxidase subunit (COX6A1). FABP6 and 1, and COX6A1 are involved in bile acid metabolism and energy metabolism, whilst ARL8B is involved in antigen presentation. The five proteins showed increased abundance in the ETEC pigs and are involved in immune response, metabolism of nuclear acid, protein and cell cycle regulation.

### 2.3. Transcription of Ileal Genes

**Figure 2:**
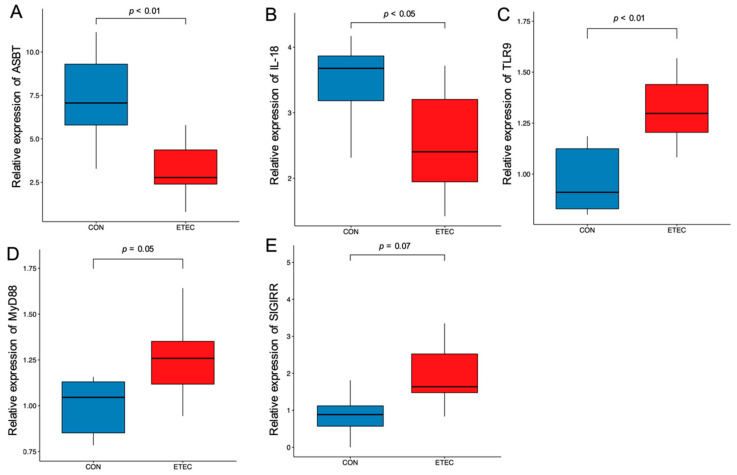
Transcription in ileum of selected genes ASBT (**A**), IL-18 (**B**), TLR9 (**C**), MyD88 (**D**), SIGIRR (**E**).

To extend the findings by proteomics, a group of selected genes was tested by RT-qPCR. Transcription levels of ASBT (*p* < 0.01) and IL-18 (*p* < 0.05) decreased, whilst those of TLR9 (*p* < 0.01), MyD88 (*p* = 0.05) and SIGIRR (*p* = 0.07) increased in the ETEC pigs, relative to the CON ones (Figure 2**)**. Transcription of the gene HIF-α (hypoxia-induced factor-α) was also documented and used for later correlation analysis.

### 2.4. Mucosal Microbiomics

**Figure 3:**
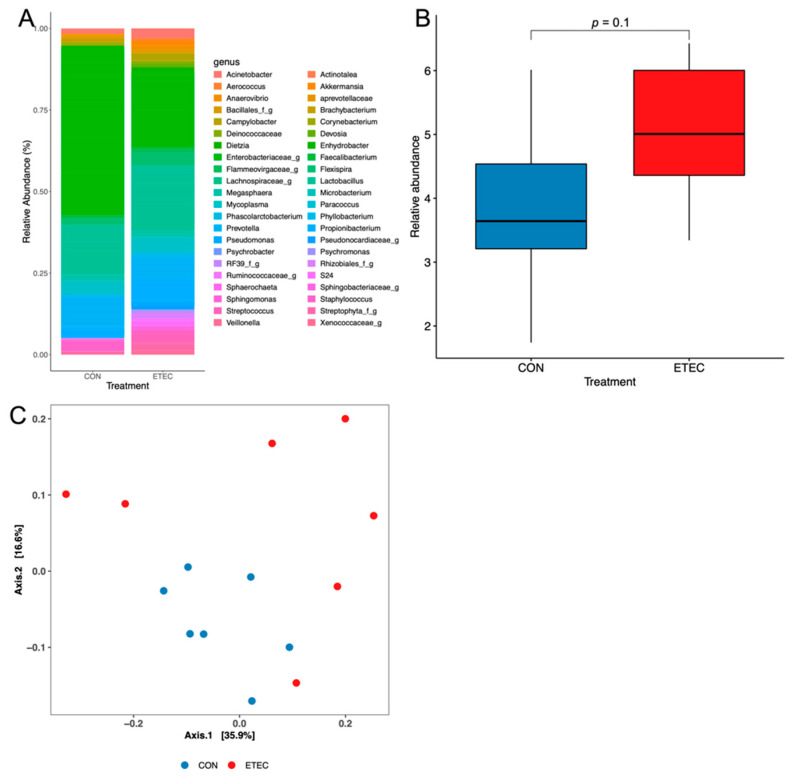
Relative richness plot (**A**); α-diversity (**B**); and β-diversity (unifrac dissimilarity) (**C**) of ileal mucosa microbiome at the genus level.

In total, 447 operating taxonomy units (OTUs) were detected. Proteobacteria and Firmicutes were the two major phyla, followed by Bacteroidetes. The Bacteroidetes: Firmicutes ratios were 0.61 and 0.54 in the ETEC and CON pigs, respectively, but no significant difference was found between the two groups. The relative richness, the alpha diversity evaluated by the Shannon diversity index and the beta diversity with unifrac distance at the genus level are shown in Figure 3. No significant difference (*p* > 0.05) was found in the alpha diversity (Wilcoxon rank-sum test, Figure 3B) or beta diversity (Adonis permutation test, Figure 3C) between the ETEC and CON groups. A genus from the order Rhizobiales showed significantly increased levels (*p* < 0.05, |effect size| = 0.62, Wilcoxon rank-sum test) in the ETEC pigs relative to the CON ones. Two unassigned genera from the families Pirellulaceae (*p* = 0.05, |effect size| = 0.62) and Enterobacteriaceae (*p* = 0.10, |effect size| = 0.62) showed trends of increase and decrease in the ETEC group, respectively.

### 2.5. Correlation of the Mucosal Microbiome and Intestinal Proteome

**Figure 4:**
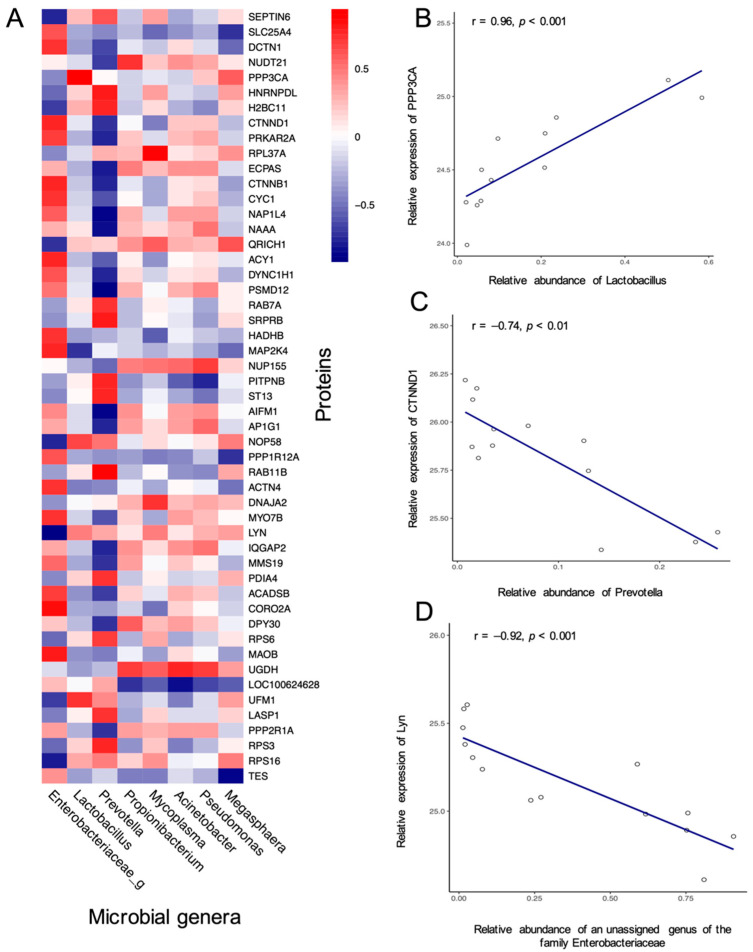
Results of the correlation analysis of the microbial genera-protein pairs. Spearman correlation heatmap of the correlation between specific bacterial genera and mucosal proteins (**A**), Spearman correlation coefficients and scatter plots of three key correlation pairs (**B**–**D**).

Regularised canonical correlation analysis (rCCA) [16] was used to explore correlations between the proteomic (proteins) and microbiome (bacterial genera) data. rCCA-selected potential correlations were further verified by Spearman’s correlation analysis. The correlation results of these proteins and bacterial genera (*p* < 0.05, r > 0.8) are presented in Figure 4. Based on the results, Figure 4B–D display the detailed relationship of three key microbial genera-protein pairs in the study. The genus Lactobacillus was positively correlated with calcineurin A (PPP3CA, r = 0.96, Figure 4B). A negative correlation was observed between the genus Prevotella and catenin δ1 (CTNND1, r = −0.74, Figure 4C). An unclassified genus of the family Enterobacteriaceae was negatively correlated with tyrosine-protein kinase Lyn (r = −0.92, Figure 4D).

## 3. Discussion

In this study, the ileal proteomic and mucosal microbiome responses to the oral challenge of ETEC were documented. As revealed by the proteomics analysis, ileal proteins involved in various biological functions were differentially regulated in the ETEC pigs, including the proteins related to immunity and infection, and to metabolisms of bile acids and energy.

Among the proteins with differential abundance, proteins involved in bile acid transport, such as FABP6 and ASBT, showed decreased expression in the ETEC pigs. Bile acid transport is of pivotal importance in the metabolism of lipids. FABP6, mainly expressed in the ileum, transports bile acids in the epithelial cells to the basolateral domains then exits the epithelial cells [17]. Similar to our findings, lowered ileal expression of FABP6 was also found in the pigs orally infected by *Salmonella typhimurium* [18], suggesting the involvement of bile acids metabolism in bacterial diarrhoea. In the enterohepatic recycling of bile acids, more than 95% of the bile acids are reabsorbed in the ileum and transported back to the liver for reuse [19,20]. Apical sodium-dependent bile acid transporter (ASBT), almost exclusively expressed in the ileum, is the main transporter that absorbs bile acids from the intestinal lumen [21]. Non-synonymous substitutions in the ASBT amino acid sequence cause bile acid malabsorption and are associated with diarrhoea [19]. In vitro studies showed that challenge with *E. coli* significantly decreased the expression of ASBT in Caco2 cells [22]. Lack of ASBT results in faecal excretion of bile acids [23]. Bile acids entering enterocytes assisted by ASBT activate the farnesoid X receptor (FXR), which activates FABP6 [24]. As reported, the decreased expression of ASBT and FABP6 demonstrated impaired bile acid absorption in the ileum possibly induced by the early inflammatory response [25]. Therefore, an accumulation of bile acids in the intestinal lumen, which is toxic to enterocytes is postulated. However, no luminal samples being collected leaves it impossible to assess the bile acids levels in the digesta.

ETEC-induced diarrhoea involves mucosal inflammation, which is at least partly mediated by TLRs, as shown in murine [10] and pig models [26]. In our study, TLR9 was increased in the ETEC pigs, which led to up-regulated transcription of MyD88 (myeloid differentiation factor 88), SIGIRR (single immunoglobulin domain-containing IL1R-related) and the down-regulated transcription of IL-18, which is in accordance with previous studies [27,28]. TLR9 recognises unmethylated CpG dinucleotides present in the bacterial DNA and triggers downstream signal pathways, leading to elevated mucosal inflammation [29,30]. Up-regulation of downstream MyD88 and SIGIRR indicated the activation of this pathway [31]. However, activation of TLR9 on the apical/luminal surface of enterocytes can also inhibit NF-κB signalling, leading to the tolerance to the stimulation of TLRs, including TLR2 and TLR4 [32]. This may have contributed to the unchanged transcription of TLR2 and 4 detected here in this study (data not shown). Further investigation is needed on the cross-talk between the different pathways under the ETEC challenge. Of note, pro-inflammatory IL-18 does not only play a part in the mucosal inflammation triggered by the ETEC challenge, but is also involved in the maintenance of the intestinal barrier [33]. Decreased transcription of IL-18 observed here may have contributed to the impaired intestinal barrier frequently observed in the ETEC-challenged pigs.

It was suggested that ETEC-induced diarrhoea is associated with an askew gut microbiome, which, not the ETEC per se, triggers the diarrhoeal symptoms [14]. ETEC attaches to the intestinal tissue by its adhesins, which promote binding and colonisation on the intestinal epithelium [5]. Studies have documented the changes of the faecal or digesta microbiome in ETEC-treated pigs [34,35,36]. In this study, the mucosal microbiome, instead of the luminal or faecal one, was profiled by direct analysis of the ileal tissues with the emptied luminal content. About 80% of the bacteria detected belong to the Proteobacteria and Firmicutes phyla, which is similar to the digesta or faecal microbiome [14]. An increased Bacteroidetes: Firmicutes ratio was found in the ETEC pigs, which is different from previous studies on pig models [14,37]. In a previous study, the effects of ETEC on the microbiome of ileal digesta, but not on that of faeces, were observed in both community richness and diversity on day 9 post-inoculation [36]. In this study, however, no significant difference was observed in relative abundance between the two treatment groups, which suggests the mucosal microbiome was relatively stable under the ETEC challenge. The exact roles of the mucosal and digesta (luminal) microbiome in response to ETEC infection need further study.

rCCA was adopted to reveal potential correlations between the intestinal proteins and mucosal bacterial taxa. The positive correlation between Lactobacilllus and PPP3CA suggests a potential role of Lactobacilllus in regulating the intestinal function, as PPP3CA induces the transcription of HIF-1α [38], which can alleviate local inflammation and maintains the intestinal barrier through the induction of intestinal trefoil factor (ITF) and Ecto-5´-nucleotidase (CD73) [39,40,41]. Similar to our findings, *Lactobacillus* was associated with HIF-responsive ITF in a mouse model [42]. Faecal levels of the genus of *Prevotella* were negatively associated with diarrhoea [14], though *Prevotella* stimulates epithelial cells to produce cytokines and promotes Th17-mediated inflammation and neutrophil recruitment [43]. CTNND1 regulates cell–cell adhesion [44]. The negative correlation found in our study suggested that mucosal *Prevotella* may affect intestinal inflammation partially through the regulation of CTNND1. Lyn is a member of the Src family kinases (SFK) regulating the response of immune cells, such as B cells and mast cells, and promotes the production of immune factors [45]. Studies have shown that bacteria from the family Enterobacteriaceae, such as *Citrobacter rodentium* and *Salmonella entericaare*, could affect the expression of Lyn [46]. Due to the limits of the sequencing technology adopted, the exact genus correlated with Lyn in the ileum was unknown and further experiments are needed.

## 4. Materials and Methods

### 4.1. Animal Procedure and Sample Collection

The animal experiment was carried out in accordance with the guidelines and regulations of the Animal Care and Ethical Committee of the Sichuan Agricultural University and was in compliance with the ARRIVE guidelines. The ethical approval for the animal procedure was granted by the Animal Care and Use Committee of the Sichuan Agricultural University (SCAUAC-20200051).

After acclimatisation for a week, 14 male weaned pigs aged 28 d (Duroc × Landrace × Yorkshire) were moved to the pig nursery house with plastic slatted floor and were randomly allocated into two groups, the group challenged by enterotoxigenic *Escherichia coli* K88 (ETEC, *n* = 7) and the unchallenged group (CON, *n* = 7). The pig nursery house contained 18 pens (1.5 × 1.5 m) with infrared lamps (250 W) hanging above the pens with lights on from 8:00 to 24:00. The ambient temperature was maintained at 26 ± 2 °C, and relative humidity was controlled at 60 ± 5%. The inoculum for the ETEC group is Luria Broth containing ETEC (100 mL, 1 × 10^9^ CFU/mL, serotype O149: K91: K88ac; China Veterinary Culture Collection Centre, Wuhan, China), and the CON group received 100 mL sterilised Luria broth. The two groups were reared on the same diet and water ad libitum, kept in pens in separate rooms to avoid cross-contamination. The ingredient composition and nutrient levels of the diet are presented in Table S1.

Self-defined diarrhoea scores (0, normal; 1, pasty; 2, semi-liquid; 3, watery) were recorded at 0, 4, 8, 16, 20, 24, 30 and 36 h after the ETEC challenge for each rearing pen. All pigs were euthanised with an intramuscular injection of 15 mg/kg body weight of pentobarbital sodium under anaesthesia 48 h after the ETEC challenge. Two sections of ileal tissue (2 cm in length) were collected from each pig with one stored in 4% paraformaldehyde solution for histology and the other snap-frozen and stored at −80 °C for proteomic and transcription analyses.

### 4.2. Intestinal Morphology

Ileal tissues were cross-sectioned and stained with the periodic acid Schiff method (PAS staining). Ten villi and crypts of each section were measured (Image ProPlus 6.0, Media Cybernetics, Rockville, MD, USA) under a microscope and the villi/crypt ratio (VCR) was calculated.

### 4.3. Proteomics of the Ileal Tissues

The ileal samples were processed according to the enhanced filter-aided sample preparation (eFASP) protocol [47]. Briefly, frozen tissue samples were homogenised using a TissueLyser II (QIAGEN, Gaithersburg, MD, USA) in a lysis buffer containing 4% sodium dodecyl sulphate, 0.2% deoxycholic acid, 50 mmol/L dithiothreitol and 100 mmol/L ammonium bicarbonate (pH 8.0). The lysate was incubated at 95 °C for 5 min and centrifuged (16,000× *g*, 4 °C, 20 min). Protein concentration of the obtained supernatant was determined by BCA protein quantification kit (Thermo Scientific, Waltham, MA, USA). Supernatant containing 100 µg protein was transferred onto a centrifugal filter (Amicon Ultra, 10 kDa, Millipore, Darmstadt, Germany) and washed twice by mixing with an exchange buffer (8 mol/L urea, 0.2% deoxycholic acid, 100 mmol/L ammonium bicarbonate, pH 8.0) followed by centrifugation (14,000× *g*, 15 min). The protein was reduced by Tris(2-carboxyethyl) phosphine (TCEP, 0.01 mol/L, 1:50 [*v*/*v*]), alkylated with iodoacetamide and digested by trypsin (1 µg/100 µg protein, Promega, Madison, WI, USA). Tryptic peptides were recovered and purified by phase extraction using ethyl acetate acidified by formic acid (1%, *v*/*v*). Vacuum-dried tryptic peptides were resuspended in 2% acetonitrile with 0.1% formic acid and applied onto a Dionex RSLC UPLC System (Thermo Scientific) coupled to an Orbitrap Fusion Lumos Mass Spectrometer (Thermo Scientific). One µg of peptide was injected onto a 2 cm C18 material-trapping column and separated on an analytical column (Acclaim PepMap100, 75 µm ID, 15 cm, 100 Å, Thermo Scientific) with both columns kept at 40 °C. The peptides were eluted at a stable flow rate of 300 nL/min with a linear gradient from a solution of 2.4% acetonitrile and 0.1% formic acid to a solution of 78% acetonitrile and 0.1% formic acid in 150 min. Mass spectrometric data were obtained in the positive ionisation mode in data-dependent acquisition (DDA) fashion. The mass spectra are available at the ProteomeXchange Consortium (proteomexchange.org) with the data set identifier PXD028066.

Protein annotation and quantification were carried out using MaxQuant (version 1.5.2.8) [48] against the Uniprot database (*Sus scrofa*, UP000008227, last modified 29 January 2021). Detection of at least two unique peptides per protein and protein being present in at least 50% of the samples in each group were required. Protein abundance data were normalised and two-based logarithm transformed using the Perseus software (version 1.6.5.0) [49] before data analysis.

### 4.4. RT-qPCR of Ileal Genes

Transcription levels of selected genes in the ileum were tested by quantitative RT-qPCR using predesigned primers (Table S2). Briefly, tissue RNA was extracted using Trizol Reagent (TaKaRa Biotechnology, Dalian, China) according to the manufacturer’s instructions. The reverse transcription was performed using a cDNA reverse transcription kit (Vazyme Biotechnology, Nanjing, China) and the obtained cDNA was amplified with an SYBR green kit (Vazyme Biotechnology, Nanjing, China) on an ABI-7900HT Fast Real-Time PCR System (Applied Biosystems, Foster City, CA, USA). The transcription levels of target genes were normalised to the housekeeping gene, ACTB, and analysed using the 2 −∆∆Ct method [50].

### 4.5. Full-Length 16S Sequencing of the Ileal Mucosal Microbiome

The full-length 16S sequencing of the ileal mucosal microbiome was performed as previously described [51]. Briefly, the ileal mucus was scraped and the bacterial genomic DNA was extracted with a DNA Stool Mini-Kit (QIAGEN, Hilden, Germany). The DNA concentration was estimated on a NanoDrop spectrophotometer (Thermo Scientific). The 16S rRNA gene was amplified by PCR with specific primers (Table S3). The DNA libraries were constructed on the amplicons with the SMRT Bell technology on a PacBio RS II sequencer (Pacific Biosciences, Menlo Park, CA, USA) [52]. The raw reads were processed using Lima software (version 1.11.0) to obtain circular consensus sequence (CCS) reads. Further sample sorting, trimming and clustering of OTUs were conducted with the USEARCH software [53].

### 4.6. Data Analysis

All data analyses were conducted in R [54] integrated with R Studio [55]. Body weight, diarrhoea score, intestinal morphology and transcription levels of ileal genes were analysed by Student’s *t*-test or Wilcoxon rank-sum test.

The proteomic data were firstly analysed with a semi multivariate method, the NSC analysis to locate proteins with differentiating power of the two treatment groups using the package *pamr* [56]. The amount of shrinkage value was determined using 5-fold cross-validation, and the NSC probability analysis with a probability cut-off of 90%. Proteins selected by NSC were further tested by Student’s *t*-test or Chi-squared test. A protein with a *p* < 0.05 and absolute value of effect size >0.8 was regarded as significantly different between the treatment groups.

For the mucosal microbiome analysis, OTUs present in at least 50% of all samples or higher than 75% of all counts were selected and a total of 111 were selected. OTU information and abundance were aligned with treatment groups for data analysis. Alpha-diversity shown as Shannon index was calculated and compared between the treatment groups. Beta-diversities based on the unifrac and weighted unifrac distances were tested by permutation test and presented in PCoA plots. Wilcoxon rank-sum test was used to analyse the taxon abundance between the treatment groups.

### 4.7. Correlation Analysis of The Microbiome and Proteomic Data

Correlation between the mucosal microbiome and proteomic data was firstly conducted by multivariate correlation analysis, rCCA, using the R package *mixOmics* [57]. The regularising λs for the two datasets were obtained by cross-validation. Any correlation with the correlation product > 1.0 was selected and was further verified with Spearman correlation analysis. Only the correlations with *p* < 0.05 and correlation coefficient (r) > 0.8 were presented.

## 5. Conclusions

The oral challenge of ETEC affected ileal proteins involved in various biological functions, such as bile acid metabolism and the NF-κB pathway. The impact of ETEC infection on the mucosal microbiome in the ileum was limited but the correlation of bacteria to ileal protein expression was observed. Further studies are needed to verify our findings on the underlying mechanism in the pathogenesis of ETEC-triggered diarrhoea.

## Figures and Tables

**Table 1:** The body weight at euthanasia and the intestinal morphological parameters.

	CON ^a^	ETEC ^a^	*p*
Body weight, kg	6.67 ± 0.36	6.06 ± 1.07	0.33
Villus height, μm	242.11 ± 95.79	236.50 ± 60.74	0.91
Crypt depth, μm	192.83 ± 29.73	219.91 ± 27.59	0.13
VCR ^b^	1.37 ± 0.74	0.98 ± 0.28	0.26

^a^ Data are shown as mean ± SD; CON, no ETEC challenge; ETEC, ETEC challenge. ^b^ The ratio of villous height over crypt depth.

**Table 2:** Proteins with differential abundance between the CON and ETEC pigs.

Accession Number	Protein Name	Gene Name	Biological Process	CON ^a^	ETEC ^a^	*p*	Effect Size
P10289	Gastrotropin	FABP6	bile acid transport	32.73 ± 0.35	31.65 ± 0.9	0.01 ^b^	1.46
P49924	Fatty acid-binding protein, liver	FABP1	fatty acid transport	28.59 ± 0.84	27.38 ± 0.91	0.03	1.28
F1SFL1	ADP ribosylation factor such as GTPase 8B	ARL8B	antigen processing and presentation	24.77 ± 0.2	24.32 ± 0.17	0.01	2.20
A0A287BGN0	Cytochrome c oxidase subunit	COX6A1	energy metabolism	26.55 ± 0.35	25.88 ± 0.33	0.01	1.80
K7GNN0	Von Willebrand factor	VWF	immune response	23.97 ± 0.29	24.47 ± 0.24	0.01	-1.74
A0A287BTC2	DNA-(apurinic or apyrimidinic site) endonuclease	APEX1	nuclear acid metabolism	25.63 ± 0.17	26.08 ± 0.18	<0.01	-2.41
A0A5G2QGY8	Tubulointerstitial nephritis antigen-like 1	TINAGL1	proteolysis	23.49 ± 0.47	24.2 ± 0.43	0.02	-1.44
F1SUH2	Cell division cycle and apoptosis regulator 1	CCAR1	cell cycle regulation	26.12 ± 0.45	26.83 ± 0.07	0.01^b^	-1.97
F1RIV0	2’-5’-oligoadenylate synthase-like protein isoform a	OASL	defense response to virus	24.34 ± 0.26	25.15 ± 0.57	0.05	-1.75

^a^ Data are 2-based logarithm transformed and shown as mean ± SD; CON, no ETEC challenge; ETEC, ETEC challenge; *p*-values are calculated by Student’s *t*-test or ^b^ Wilcoxon rank-sum test.

## Data Availability

The data presented in this study are available on request from the corresponding author.

## Supplementary Materials

The following are available online at https://www.mdpi.com/article/10.3390/pathogens11020160/s1, Table S1: The ingredient composition and nutrient levels of the diet; Table S2: Predesigned primers of selected genes tested by quantitative RT-qPCR; Table S3: Primers used to amplify 16S rRNA gene.
