# Supplementary material for: Intestinal and Mucosal Microbiome Response to Oral Challenge of Enterotoxigenic Escherichia coli in Weaned Pigs
Source: Pathogens. 2022 Jan 26;11(2):160. doi: 10.3390/pathogens11020160 (PMC8879466; doi:10.3390/pathogens11020160)
Supplement: Supplementary file 1 [file pathogens-11-00160-s001.zip › pathogens-1535701-supplementary.pdf]

Table S1. The ingredient composition and nutrient levels of the diet.

Ingredients	Percentage (%)
Corn	51.59
Dehulled soybean meal (46%CP)	16.00
Fermented soybean meal	10.00
Deproteinized whey powder（2%CP）	8.00
Fish meal (62.5 %CP)	2.50
Formula	2.00
Soyabean oil	2.00
Sucrose	2.00
Glucose	2.00
L-Lys.HCL (98%)	0.58
DL-Met (98.5%)	0.25
L-Thr (98%)	0.22
L-Trp (98%)	0.05
Choline chloride (50%)	0.16
$CaCO_3$	1.00
$CaHPO_4$, 17/21	0.80
Salt	0.40
Zinc Oxide	0.20
Mineral mixture†	0.20
Vitamin mixture‡	0.05
Total	100.00
Nutrient Level	
Digestible energy (Mcal/kg)	3.56
Crude protein %	19.74
Calcium %	0.80
Total phosphorus %	0.57
Available phosphorus %	0.41
SID Lys %	1.43
SID Met %	0.54
SID Met + Cys %	0.78
SID Thr %	0.83
SID Trp %	0.22

CP crude protein; SID standard ileal digestibility

† the mineral premixture provided the following minerals per kg of complete diet: Fe, 100 mg; Cu,150 mg; Zn, 100 mg; Mn, 20 mg; iodine, 0.3 mg; Se, 0.3 mg.

‡ the vitamin premixture provided the following vitamins per kg of complete diet: Vitamin A,

9000IU; Vitamin D3, 3000IU; Vitamin E, 20IU; Vitamin K3, 3.0mg; Vitamin B1, 1.5mg; Vitamin B2, 4.0mg; Vitamin B6, 3.0mg; Vitamin B12, 0.2mg; nicotinic acid, 30.0 mg; pantothenic acid, 15.0 mg; folic acid, 0.75 mg; biotin, 0.1 mg.

Table S2. Predesigned primers of selected genes tested by quantitative RT-qPCR.

Gene		Primer (5'–3')	Product length (bp)	Gene Bank accession
ASBT	Forward	AGGTTGGATCCATCACAGGC	134	NM_001244463.1
	Reverse	GGAGTAACCGGCCAAAGGAA		
IL-18	Forward	AGCTGAAAACGATGAAGACCTG	121	NM_213997.1
	Reverse	AAACACGGCTTGATGTCCCT		
TLR-9	Forward	AATCCAGTCGGAGATGTTTGCT	79	AY859728
	Reverse	GACCGCCTGGGAGATGCT		
MyD88	Forward	GTGCCGTCGGATGGTAGTG	65	NM001099923
	Reverse	TCTGGAAGTCACATTCCTTGCTT		
SIGIRR	Forward	ACCTGGGCTCCCGAAACTAC	62	AK239384.1
	Reverse	GTCATCTTCTGACACCAGGCAAT		
β-actin	Forward	GGCGCCCAGCACGAT	66	DQ845171.1

Table S2. Continued.

Gene		Primer (5'–3')	Product length (bp)	Gene Bank accession
β-actin	Reverse	CCGATCCACACGGAGTACTTG		

ASBT apical sodium-dependent bile acid transporter; IL-18, interleukin-18; TLR9 Toll-like receptor9; MyD88, myeloid differentiation factor 88; SIGIRR, Single Ig And TIR Domain Containing; HIF, hypoxia inducible factor.

Table S3. Primers used to amplify 16S rRNA gene.

Sample ID		Primer (5'–3')
CON 1	Forward	ACAGTCGAGCGCTGCG
	Reverse	GTATGTGAGAGAGCGC
CON 2	Forward	ACACTAGATCGCGTGT
	Reverse	GTATGTGAGAGAGCGC
CON 3	Forward	CACGACACGACGATGT
	Reverse	CACGCGACGCTCTCTA
CON 4	Forward	CGCATGACACGTGTGT
	Reverse	GTATGTGAGAGAGCGC
CON 5	Forward	CACTCACGTGTGATAT
	Reverse	CACGCGACGCTCTCTA
CON 6	Forward	CACGACACGACGATGT
	Reverse	GTATGTGAGAGAGCGC
CON 7	Forward	CATGTAGAGCAGAGAG
	Reverse	CACGCGACGCTCTCTA
ETEC 1	Forward	CACTCACGTGTGATAT

Table S3. Continued.

	Reverse	GTATGTGAGAGAGCGC
ETEC 2	Forward	CATGTAGAGCAGAGAG
	Reverse	GTATGTGAGAGAGCGC
ETEC 3	Forward	CACTCGACTCTCGCGT
	Reverse	CACGCGACGCTCTCTA
ETEC 4	Forward	TCTGTATCTCTATGTG
	Reverse	CACGCGACGCTCTCTA
ETEC 5	Forward	ACAGTCGAGCGCTGCG
	Reverse	CACGCGACGCTCTCTA
ETEC 6	Forward	ACACTAGATCGCGTGT
	Reverse	CACGCGACGCTCTCTA
ETEC 7	Forward	CGCATGACACGTGTGT
	Reverse	CACGCGACGCTCTCTA
